# Diatom volatile organic compound production is driven by diel metabolism and the cell cycle

**DOI:** 10.3389/fmicb.2025.1620542

**Published:** 2025-10-06

**Authors:** Vaishnavi G. Padaki, Emily Palmer, Yuan Jiang, Holger H. Buchholz, Jeffrey A. Kimbrel, Kimberly H. Halsey

**Affiliations:** ^1^Department of Microbiology, Oregon State University, Corvallis, OR, United States; ^2^Department of Statistics, Oregon State University, Corvallis, OR, United States; ^3^Lawrence Livermore National Laboratory, Livermore, CA, United States

**Keywords:** volatile organic compounds, diel VOC metabolism, phytoplankton physiology, cell cycle, central carbon metabolism

## Abstract

**Introduction:**

Volatile organic compounds (VOCs) are small, low-vapor-pressure molecules emitted from the surface ocean into the atmosphere. In the atmosphere, VOCs can change OH reactivity and condense onto particles to become cloud condensation nuclei. VOCs are produced by phytoplankton, but the conditions leading to VOC accumulation in the surface ocean are poorly understood.

**Methods:**

In this study, VOC accumulation was measured in real time over a 12 h day−12 h night cycle in the model diatom *Phaeodactylum tricornutum* during exponential growth.

**Results:**

Sixty-three *m/z* signals were produced in higher concentrations than in cell-free controls. All VOCs, except methanol, were continuously produced over 24 h. All VOCs accumulated to higher concentrations during the day compared to the night, and 11 VOCs exhibited distinct accumulation patterns during the morning hours. Twenty-seven VOCs were associated with known metabolic pathways in *P. tricornutum*, with most VOCs involved in amino acid and fatty acid metabolism.

**Discussion:**

Patterns of VOC production were strongly associated with diel shifts in cell physiology and the cell cycle. Diel VOC production patterns give a fundamental understanding of the first steps in VOC accumulation in the surface ocean.

## Introduction

Phytoplankton are the primary source of the diverse array of labile dissolved organic compounds (DOC) in the surface ocean (Hansell, 2013; Moran et al., 2022), where DOC concentrations are maintained at 10–30 μM via uptake and metabolism by heterotrophic bacterioplankton (Hansell, 2013). Volatile organic compounds (VOCs) are chemicals with relatively low molecular weight and high vapor pressures that can comprise up to 30% of DOC (Hansell, 2013; Ruiz-Halpern et al., 2014). VOC physicochemical properties allow them to readily diffuse out of phytoplankton cells into seawater (Achyuthan et al., 2017; Zuo, 2019), where they can be taken up by bacterioplankton (Dawson et al., 2021; Moore et al., 2022; Simó et al., 2022), photochemically oxidized (Kieber et al., 1990; De Bruyn et al., 2020), or emitted into the atmosphere where they impact climate (Lelieveld et al., 2009). VOC air-sea emissions alter atmospheric chemistry and can influence the formation of secondary organic aerosols (SOA) (Quinn and Bates, 2011; Liu and Matsui, 2022) and cloud condensation nuclei (Charlson et al., 1987; Jackson et al., 2020; Mayer et al., 2020; Meskhidze and Nenes, 2006). The roles of VOCs in the marine microbial carbon cycle and climate highlight the need to understand the metabolic processes underlying VOC accumulation in the surface ocean.

VOC production is often associated with phytoplankton stress, which can occur when cells are exposed to light intensities or nutrient concentrations that are much higher or lower than the cells' acclimation status (Zuo et al., 2012; Meskhidze et al., 2015; Wang et al., 2021). For example, the well-studied VOCs, DMS and isoprene, are thought to provide antioxidant properties under high light stress (Zuo, 2019; Hackenberg et al., 2017; Dani et al., 2020; Fisher et al., 2020), iron limitation and UV exposure (Sunda et al., 2002), and nitrogen limitation (Sunda et al., 2007). Production of benzene and xylene was stimulated by ozone exposure in seawater communities, and those VOCs were suggested to protect cells from oxidative damage (Rocco et al., 2021). DMS is also produced by phytoplankton under grazing pressure (Fink, 2007; Fisher et al., 2020; Pozzer et al., 2022) and has recently been shown to serve as an info-chemical that attracts grazers to their phytoplankton prey (Shemi et al., 2023), potentially causing a positive feedback loop. The microalga, *Microchloropsis salina*, produced several volatile hydrocarbons that could serve as biomarkers of its grazing by a marine rotifer (Fisher et al., 2020). During harmful cyanobacterial algal blooms, oxidative stress in a freshwater lake caused saturated fatty aldehydes to increase in concentrations (Collart et al., 2023). Methanol production was highest at the onset of the stationary phase in cultures of phytoplankton from a range of taxonomic groups, suggesting nutrient limitation and cell envelope restructuring trigger methanol release (Mincer and Aicher, 2016), similar to methanol production associated with pectin demethylation in higher plants (Fall and Benson, 1996).

VOC concentrations are about ten-fold higher during optimal, non-stressed phytoplankton growth than during stationary phase (Shaw et al., 2003; Padaki et al., 2024) and may be a significant component of gross carbon production (Moore et al., 2020). Ethane, propane, and hexane increased in parallel with chlorophyll concentrations in diatom and dinoflagellate cultures (McKay et al., 1996) and in seawater (Ratte et al., 1993; Broadgate et al., 1997; Kameyama et al., 2009), suggesting their production by healthy, actively growing phytoplankton. Isoprene production rates were variable in a wide range of actively growing phytoplankton taxa (Colomb et al., 2008; Bonsang et al., 2010; Shaw et al., 2010) and correlated with photosynthetic activity in diatoms (Dani et al., 2020). In terrestrial plants, VOCs help maintain plant homeostasis during daily light fluctuations (Vivaldo et al., 2017; Liu et al., 2023) and are differentially produced depending on environmental stimuli (Broz et al., 2010; Mccormick et al., 2014). Some VOCs, such as acetaldehyde, acetone, and formaldehyde, are metabolic intermediates in biochemical pathways (Dudareva et al., 2013; Moore et al., 2020) active during normal growth. Acetaldehyde is both a product and a substrate in multiple pathways involving fatty acids, amino acids, carbohydrates, and precursors of some nucleic acids (Lees and Jago, 1978; Mazeed et al., 2021), which may explain its abundance in some VOC assays (Halsey et al., 2017). VOCs that are secondary metabolites, such as eugenol, and the isomers estragole and anethole, are antioxidants produced in healthy, growing cells (Wang et al., 2021; Rico et al., 2024).

Phytoplankton photosynthesis and cell division exhibit periodicity across the light-dark diel cycle (Bruyant et al., 2005; Mella-Flores et al., 2012; Su et al., 2024) and are tightly aligned with changes in the cell cycle (Su et al., 2024; Halsey et al., 2013). Phytoplankton cell cycles include three main phases: G1 encompasses most of the cell's biosynthetic activities and therefore spans most of the daytime (Brzezinski, 1992; Huysman et al., 2014). The S phase, or DNA synthesis, typically commences before dusk, while the G2-M phase, in which the cell replicates its organelles and divides into daughter cells, occurs during the night (Kim et al., 2017; Brzezinski, 1992). Photosynthesis increases intracellular metabolite concentrations during G1 to facilitate carbon precursors available for cell growth and division (Prézelin, 1992; Uchimiya et al., 2022). DOC in the surface waters of Suruga Bay, Japan, was often higher in concentration during the day compared to pre-dawn (Shinomura et al., 2005). These differences may be caused by phytoplankton primary production surpassing heterotrophic bacterial uptake of DOC (Shinomura et al., 2005). Daytime increases in DOC suggest that VOC production may also vary in concert with the diel cycle. During summertime in the North Atlantic Ocean, net VOC production (i.e., the rate of VOC production minus the rate of biological VOC losses) of methanol, DMS, and methanethiol were highest at midday (Davie-Martin et al., 2020). Isoprene and methanethiol exhibited clear diel variation in the diatom, *Thalassiosira pseudonana* (Kameyama et al., 2011), and higher day vs night concentrations of DMS, isoprene, and halogenated VOCs in a coral reef were attributed to biogenic and photochemical processes (Masdeu-Navarro et al., 2024).

Daytime accumulation of VOCs in the surface ocean would increase the likelihood of air-sea emissions if the accumulating VOCs are not consumed by other biological and chemical sinks (Halsey and Giovannoni, 2023). Therefore, the association of individual VOCs to the metabolic pathways from which they are produced is important to understanding the relationships between VOC production and air-sea emissions.

Diatoms contribute up to 40% of marine primary production (Field et al., 1998) and are known to produce a wide array of VOCs (Moore et al., 2020; Rocco et al., 2021). Here, we investigated diel variation in VOC production in the model diatom, *P. tricornutum* CCMP 2561. Cells were fully acclimated to sinusoidal light that mimicked the radiation in the surface ocean. VOCs were collected in real-time using proton transfer reaction time of flight mass spectrometry (PTR-TOF MS). A wide array of VOCs was detected, with VOC concentrations per cell peaking during the light phase. Many of the detected VOCs were associated with metabolic pathways to help explain their production patterns in the context of diel and cell cycle physiology. Knowledge of light-driven VOC production by phytoplankton is important to inform mechanisms of VOC accumulation in the surface ocean and their potential for air-sea VOC emissions.

## Materials and methods

### Culture growth conditions

**Figure 1:**
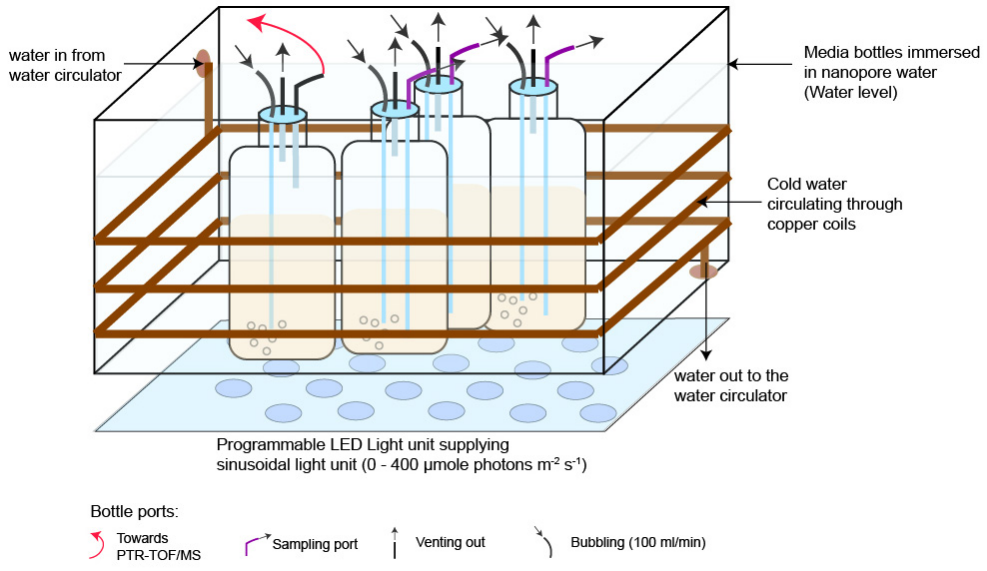
The diel VOC experimental setup with four 1-L cultures of axenic *P. tricornutum*. One culture was designated for real-time VOC measurements over 24 h (marked with a red arrow), and the remaining three were used for supporting measurements of cell counts, Chl-*a*, and photosynthetic efficiency. Temperature was maintained at 19 +/- 0.5 °C using copper coils circulating cold water around the acrylic incubator. Cultures were bubbled with breathing-grade air at 100 ml min^−1^. This setup was repeated four times with axenic *P. tricornutum* and four times with ASW-only controls.

Axenic *P. tricornutum* CCMP 2561 was cultivated under a sinusoidal 12 h:12 h light: dark cycle (0 to 400 μmole photons m^−2^ s^−1^) in f/2+Si artificial seawater medium (ASW) (Guillard and Ryther, 1962). Axenicity was confirmed prior to and during diel experiments by staining culture samples daily using SyBr Green, and fluorescence distributions analyzed by GUAVA flow cytometer (Millipore; Billerica, MA, USA) for any indication of bacterial contaminants <3 μm. Second, culture samples were analyzed prior to each diel experiment via fluorescent microscopic examination of DAPI-stained cells. The culture growth light was delivered by a programmable phyto-panel composed of white and blue light-emitting diodes (LEDs) (Photo Systems Instruments). Irradiance was measured with a 4π spherical quantum meter (Biospherical Instruments QSL-100). Cultures were maintained at 19 +/- 0.5 °C in an acrylic incubator with submerged copper tubing connected to a recirculating water bath (Figure 1).

### Diel experimental setup

For each of four replicated experiments, the incubator held four *P. tricornutum* cultures (0.6 L) in 1-L polycarbonate Nalgene^®^ Bottles. One culture was dedicated to VOC measurements, and the remaining three were used for supporting measurements (Figure 1). Abiotic control experiments (*n* = 4), in which the bottles contained only sterile ASW, were conducted after each of the four replicated culture experiments. Abiotic controls were conducted in bottles and with tubing identical to the experimental cultures. Experimental and abiotic control cultures were aerated with Ultra Zero Grade Air, and the headspace was vented through 0.2-μm Polytetrafluoroethylene (PTFE) filters (Figure 1). Cultures were kept in the exponential growth phase, using semi-continuous culturing, under these conditions for 2 weeks and then used as inocula for the diel VOC experiment. ASW prepared less than 2 days prior to initiating the diel VOC experiment was aerated for at least 6 h to remove dissolved VOCs originating from lab air and then inoculated with the axenic culture or used for the abiotic control. VOC measurements were initiated when cell densities reached 2.25–4.5 x10^5^ cells mL^−1^.

### Supporting measurements

Supporting measurements were collected every 2 h from culture samples pulled from ports in the bottle caps. Cell densities were measured by GUAVA flow cytometer (Millipore; Billerica, MA, USA). Chlorophyll concentration (Chl*a*) was determined by filtering 2–5 mL of culture (GF/F, Whatman, 25 mm), extracting in 5 mL 90% acetone at −20 °C for 24 h, and then measuring by spectrophotometer (Shimadzu; Kyoto, Japan) as described in Ritchie (2006). Photosynthetic efficiency (F_v_/F_m_) was measured by a fast repetition-rate fluorometer (FRR) following 10 min dark acclimation (Kolber et al., 1998).

### Proton transfer reaction time of flight mass spectrometry (PTR-TOF MS)

Real-time VOC measurements were initiated by removing the filter from the vent line and attaching the line directly to the PTR-TOF MS inlet. VOCs were measured via soft ionization with H_3_O^+^. The mass spectra (30–240 atomic mass units) were acquired in real-time for 24 h and then binned into 2-h intervals to match up with supporting measurements. The first 15 min in the first hour of the 24 h acquired data were removed from the analysis because of the lag time between the culture headspace and detector. PTR-TOF MS data were analyzed using PTR viewer 3.4.3 (Ionicon Analytik). Raw data in .h5 format were first calibrated against ions of known mass and internal standards (nitrosonium, NO+, at *m*/*z* 29.998 and 1,3-diiodobenzene at *m*/*z* 203.943 and *m*/*z* 330.848) using the following criteria: 3-point calibration mode, cycle 350, 0.2 *m*/*z* search range, and three spectra averaging. The accuracy of the mass calibration was confirmed against the primary ions (hydronium ions, ${\text{H}}_{3}^{18}$O+ at *m*/*z* 21.022, and H_2_${\text{O.H}}_{3}^{18}$O+ at *m*/*z* 39.035). In the PTR Viewer 3.4.3 software, each .h5 file shows an RMS error ranging from 0.006 to 0.008.

The mass spectra were visualized in the PTR-Viewer 3.4.3 (Ionicon Analytik) using the Gaussian approach, ion-mass percentage, and correctness percentage. A peak table previously collected from *P. tricornutum* (Padaki et al., 2024) was used as a reference to select *m*/*z* signals. Each peak (*m*/*z* signal) was tentatively identified using GLOVOCS (Yáñez-Serrano et al., 2021) and the ChemSpider database (integrated into PTR Viewer 3.4.3). Integrated peak signals were normalized to H_3_O^+^ concentration. Isotopic signatures of *m*/*z* signals (M) with known chemical formulas were assessed for M+1 (^13^C- and ^15^N-containing VOCs) and M+2 (^18^O- and ^34^S-containing VOCs) isotope peaks. M+1 and M+2 peak concentrations were added to M concentrations. M+1 peaks were detected for the hydrocarbons, benzene, toluene, and ethylbenzene/xylenes, C_3_H_6_, C_9_H_10_. Both ^13^C and ^15^N M+1 isotope peaks were detected for acetonitrile. The M+2 peak was detected for acetaldehyde. Detected analyte concentrations (ppbv) were calculated using the simple-reaction-kinetics approach with 30% accuracy for *E/N* values >100Td (*E/N* for this study = 126Td) (Holzinger et al., 2019).

Paired t-tests were used to identify *m*/*z* signals in each culture that were present in higher concentrations than the abiotic controls (n = 4). Benjamini-Hochberg procedure was used to control the false detection rate of *m*/*z* signals, with q-values >0.1 removed from further analysis. *m*/*z* signals from each replicate were adjusted by subtracting the corresponding *m*/*z* signal measured in abiotic controls. The adjusted *m*/*z* signals were averaged and then converted from ppbv to molar concentrations using the formula:


$$C^{*}=\frac{\left(C-B\right)\cdot Q\cdot p\cdot t}{V\cdot R\cdot T}$$

Where C^*^ is the molarity of the analyte in the culture, *C-B* is the adjusted analyte mixing ratio (ppbv), *Q* is the flow rate of air bubbled through the culture (3 L h^−1^), *p* is the atmospheric pressure (1 atm), *t* is the time period of collection (1.75 h for the first time period and 2 h thereafter), *V* is the volume of the culture (0.6 L), *R* is the gas constant (0.0821 L atm K^−1^ mol^−1^), and *T* is the incubated culture temperature (292 K). Daily production rates for each *m*/*z* signal were determined by summing the concentrations (fmol per cell) over 12 time points, i.e., the 24h period.

### Statistics

Statistically significant differences in peaks were analyzed using *t*-tests and q-values. Figures were created in R Studio 2024.12.1 + 563, R v.4.1.1, utilizing the fda, stats, ggplot2, and ComplexHeatmap packages (Gu et al., 2016; Gu, 2022), and the VOLCALC R-package (Meredith et al., 2023). The arrangement of figure panels was completed in Adobe Illustrator.

### B-spline curve fits

A smooth curve was fit for the temporal change in the mean concentrations of each peak via a regression spline function. To this end, a set of B-Spline basis functions were generated using the timepoints of the 24-h cycle. A linear combination of the B-spline basis functions gave a smooth curve to fit the mean concentrations of each peak. The coefficients of the above linear combination were estimated by balancing a goodness-of-fit criterion and a smoothness penalty to avoid underfitting or overfitting. Spline regression allows the incorporation of local and non-linear effects with a continuous covariate, which was observed in the temporal change in the peak concentrations. In this application, a B-spline basis function of the 3^rd^ order with 3 internal knots was used, resulting in 6 estimated B-spline coefficients per peak. Subsequent cluster analysis was performed using K-means on the six estimated B-spline coefficients to form groups of peaks with similar temporal changes in their mean concentrations (Supplementary Figure 1).

### Integrating *m*/*z* putative IDs to CHEBI IDs, KEGG, and RVIs

All *m*/*z* identifications extracted from the GLOVOCs database were associated with their ChEBI identifiers from the Chemical Identities of Biological Interest Database (ChEBI) (Hastings et al., 2012). The ChEBI identities were used to locate metabolites in the Kyoto Encyclopedia of Genes and Genomes (KEGG) databases for *P. tricornutum* or plants (Kanehisa, 2000; Kanehisa et al., 2016). Finally, ChEBI identities were input into the VOLCALC R-package (Meredith et al., 2023) to determine their relative volatility indices (RVIs).

## Results and discussion

### Cell physiology

**Figure 2:**
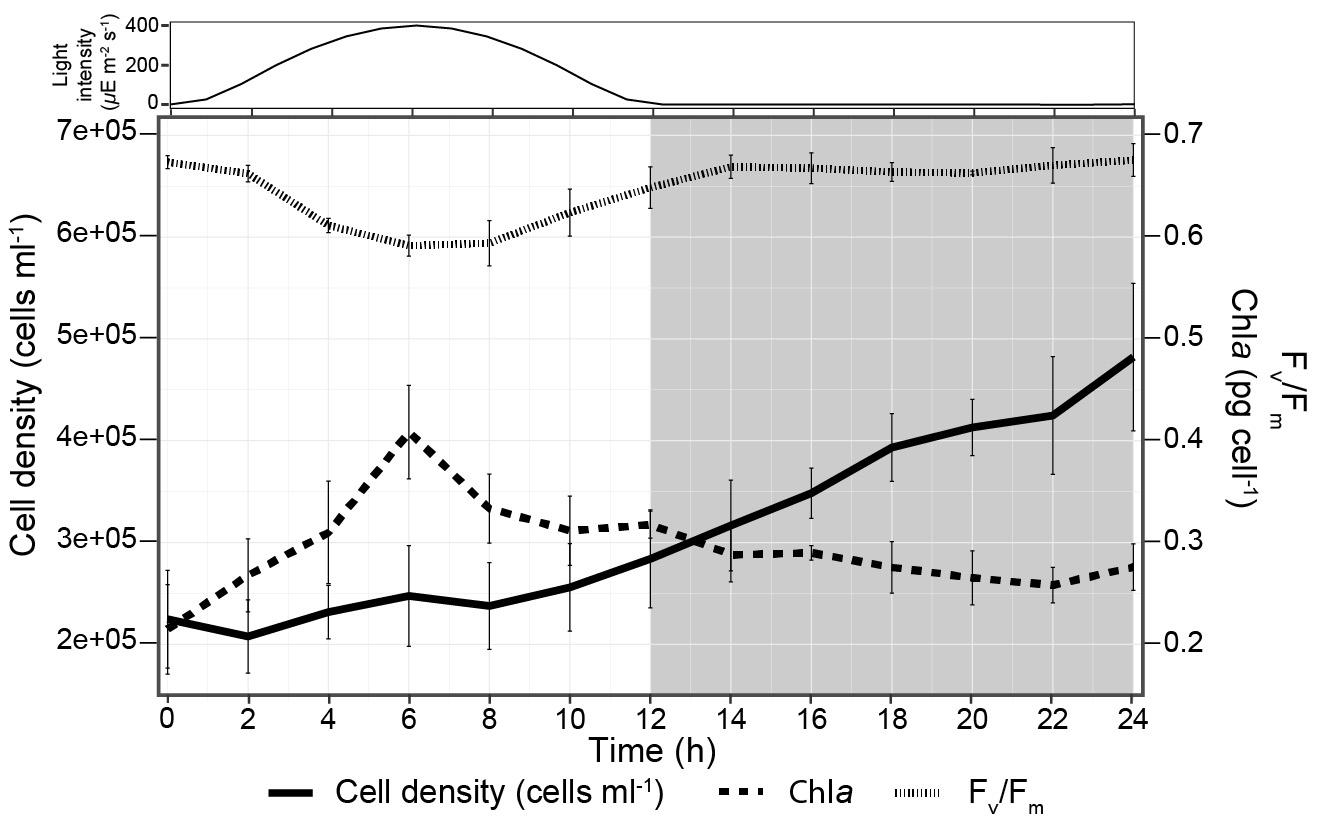
Cell physiology of *P. tricornutum* during the 24 h diel experiment. Light intensity is shown at the top, and the shaded area from 12-24 h corresponds to the night.

*P. tricornutum* grown on a 12 h:12 h light:dark sinusoidal regime exhibited a specific growth rate of 0.84 ± 0.08 d^−1^, consistent with exponential growth. The cell density stayed constant from dawn to 2 h prior to dark and then increased 2.1-fold over the remaining 14 h (Figure 2). Cell Chl*a* content doubled from dawn to midday, reaching 0.41 ± 0.04 pg cell^−1^ and then slowly decreased to the initial amount by dawn. F_v_/F_m_ was maximal (0.67 ± 0.01) at dawn and during the night but decreased at higher light intensities, consistent with photoinhibition during the midday (Domingues et al., 2012; Jallet et al., 2016) (Figure 2).

### Temporal changes in VOCs in *P. tricornutum* cultures

**Figure 3:**
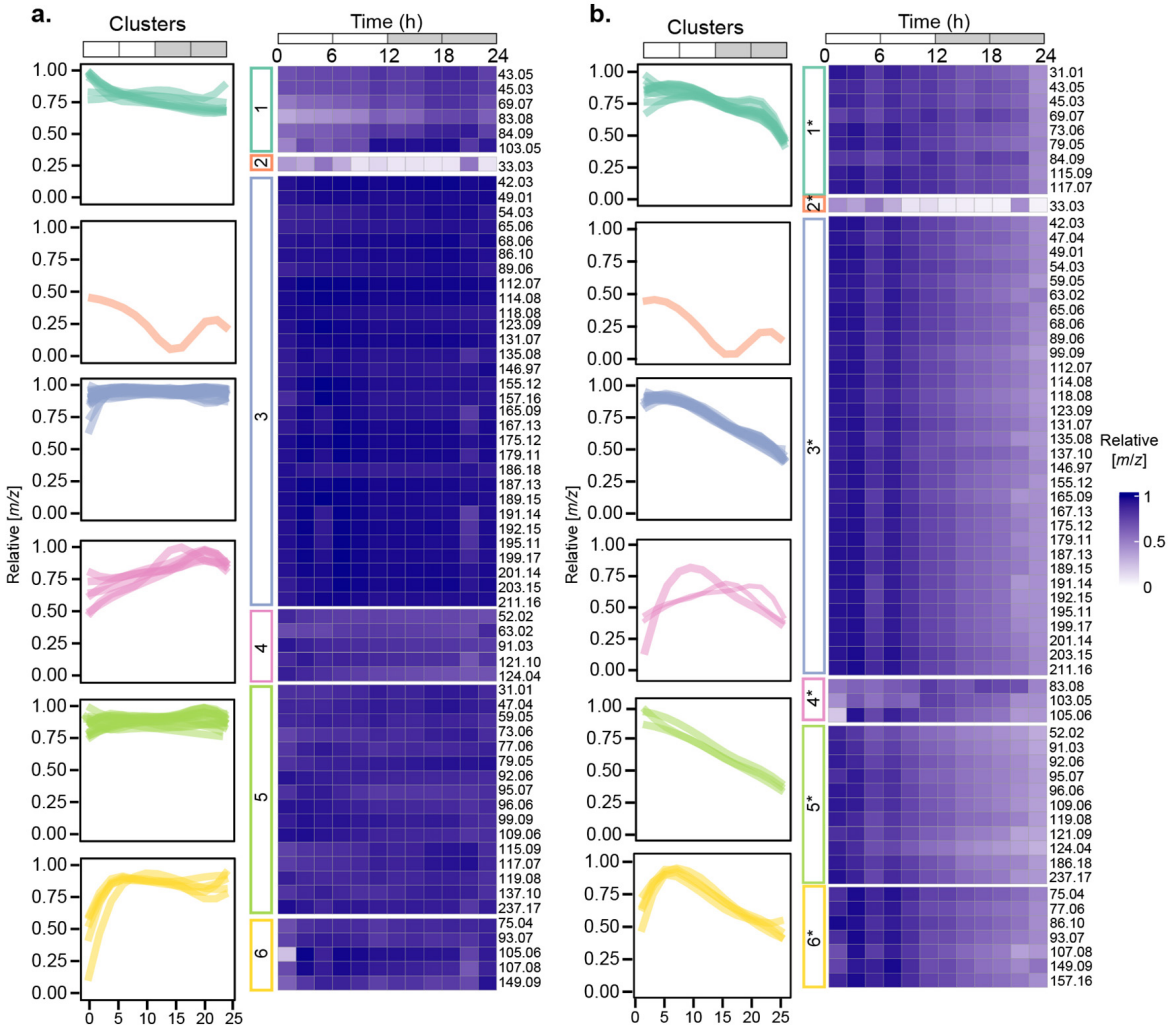
Temporal changes in relative VOC concentrations in *P. tricornutum* cultures. **(a)** Heatmaps showing concentrations of *m*/*z* signals normalized to their maximum values (rows) or **(b)**, concentrations of *m*/*z* signals normalized to cell density and then to their maximum values (rows). Relative concentrations ranged from low (light purple) to high (dark purple). Temporal patterns of *m*/*z* signal concentrations clustered into 6 different groups are shown in the leftmost column of each heatmap. *n* = 4 independent replicated cultures of *P. tricornutum*.

A total of 63 *m*/*z* signals were detected in the *P. tricornutum* cultures. Sixty-two of these signals were present at concentrations higher than media controls throughout the diel cycle, and one, *m*/*z* 33.03, corresponding to methanol, was only detected at concentrations higher than the controls from 0-8 h (Figure 3, FDR_B − H_ <0.1, *n* = 4).

To explore the data for temporal variations in VOC accumulation, we normalized the concentration of each *m*/*z* signal collected in real time during each 2 h interval in each of the four culture replicates to its maximum concentration over the 24 h experiment, and then took the average of the four replicates for each 2 h time period (Figure 3a). To decipher differences in the temporal patterns of each VOC, we fit a B-spline function with three internal knots positioned at 6, 12, and 18 h to the concentration data. These positions were selected based on the major changes in cell physiology associated with F_v_/F_m_ and cell division (Figure 1). Spline regression was used to fit curves, using third-order B-spline basis functions. The resulting curves were grouped into six clusters using K-means on their respective estimated B-spline coefficients (Figure 3a, Supplementary Figure 1).

VOCs in three clusters (1, 3, 5) were maintained at relatively constant levels throughout the diel cycle, albeit with slight variations in the early morning (Cluster 3), midday (Cluster 1), or late night (Cluster 5). Forty-six of the 63 *m/z* signals were in Clusters 3 and 5 (Table 1). VOCs in the remaining three clusters (2, 4, 6) exhibited a wider range of variation over the 24 h cycle. Cluster 2, consisting of only methanol (*m/z* 33.03), was highest at dawn and early morning and decreased to zero during the early night (Figure 3a). Cluster 4 increased from dawn until 6 h into the night. VOCs in Cluster 6 increased from dawn to midday and then remained at relatively constant levels through the night (Figure 3a).

**Table 1:** Distribution of *m*/*z* signals in each cluster identified using B-spline coefficients and K-means clustering.

**Clusters**	**Number of m/z signals**	**Clusters (normalized to cell density)**	**Number of m/z signals (normalized to cell density)**
1	6	1^*^	9
2	1	2^*^	1
3	30	3^*^	32
4	5	4^*^	3
5	16	5^*^	11
6	5	6^*^	7
Total	63		63

Clusters numbers with no asterisks are m/z signals grouped according to their temporal concentrations as shown in Figure 3a. Cluster numbers with asterisks (*) are m/z signals grouped according to their cell-specific temporal concentrations as shown in Figure 3b.

To evaluate cell-specific patterns in VOC concentrations over the diel cycle, the concentration of each *m*/*z* signal in each of the four culture replicates was divided by the cell density at each time point and then normalized to the maximum value over the 24 h period. The same 3-knot B-spline regression framework was used to fit curves for each *m*/*z* signal, and the *m*/*z* signal curves were again grouped into 6 clusters using K-means on their respective estimated B-spline coefficients, designated as Clusters 1^*^ - 6^*^ (Figure 3b). Fifty-one *m*/*z* signals were grouped into the same clusters as the un-normalized clusters (Figure 3a), and 12 *m*/*z* signals were grouped into different clusters (Figure 3, Table 1). Cluster 3^*^ included 32 *m*/*z* signals, and 52 of the 63 *m*/*z* signals were categorized into Clusters 1^*^, 3^*^, or 5^*^.

**Figure 4:**
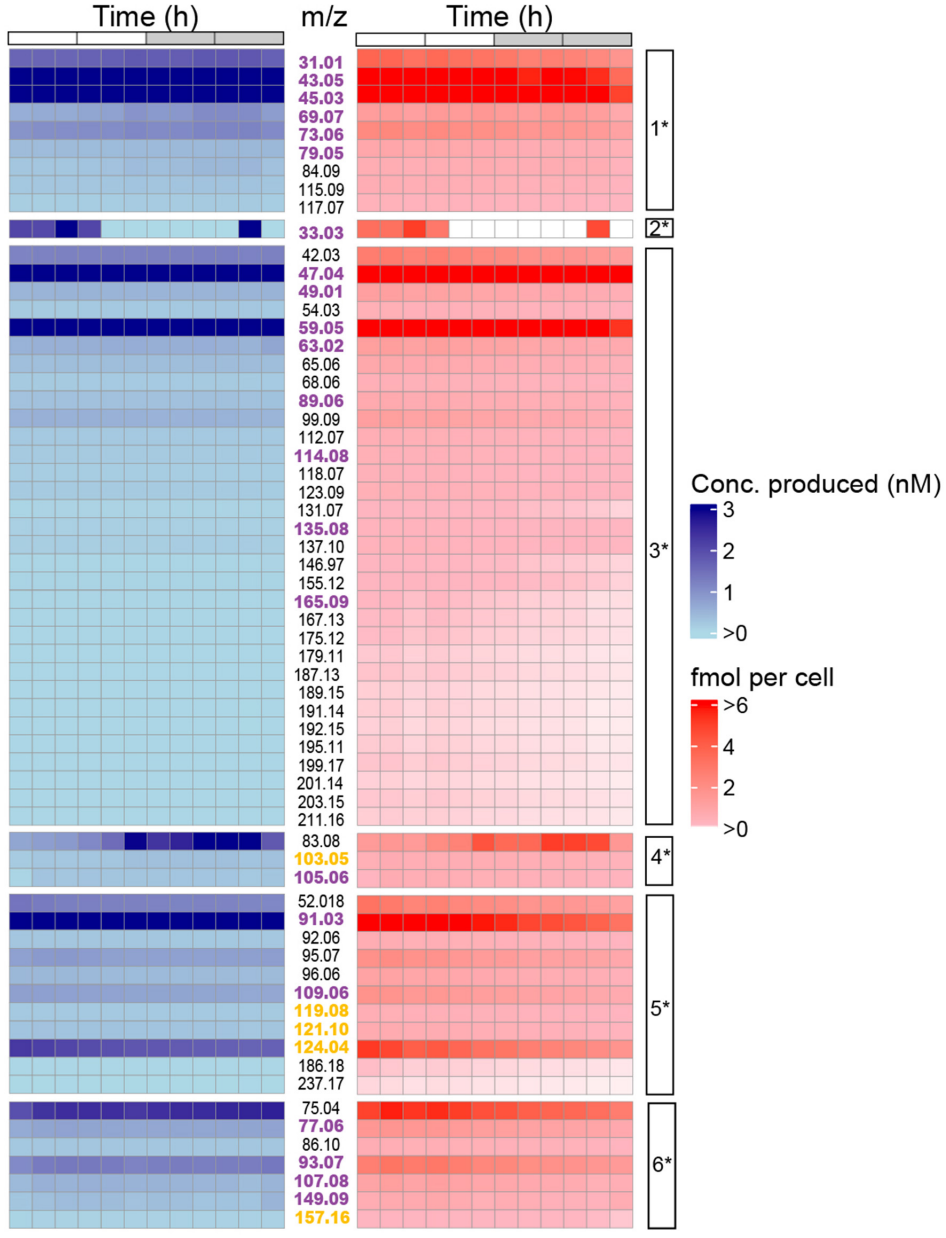
Temporal changes in absolute concentrations of *m*/*z* signals (rows) over the diel cycle in *P. tricornutum*. **(left)** Heatmap showing concentrations of *m*/*z* signals in the cultures ranging from low (light blue) to high (dark blue), and **(right)** heatmap showing cell-specific concentrations of *m/z* signals in the cultures ranging from low (light red) to high (dark red). *m*/*z* values are shown in the column between the two heatmaps. A total of 27 *m*/*z* values were found *P. tricornutum* KEGG pathways (magenta font) or plant KEGG (yellow font) pathways. The cell-specific clusters (1* - 6*) into which the *m*/*z* values were grouped are shown in the rightmost column.

Relative VOC concentrations in Clusters 1^*^, 3^*^, and 5^*^ decreased by approximately half over the diel cycle, reflecting increasing *P. tricornutum* cell density over the course of the experiment. Cluster 1^*^ showed two slight peaks at 6–8 h and 18–20 h. Cluster 3^*^ was maximal from 0–6 h, after which relative concentrations gradually decreased over the remaining 24 h. Cluster 5^*^ decreased steadily throughout the 24 h period (Figure 3b). The patterns exhibited by Clusters 2 and 2^*^, which consisted only of *m*/*z* 33.03, methanol, were independent of cell density. Methanol temporal dynamics were dominated by the changes in its concentrations, which showed strong signals between 0 and 8 h and ranged from 0.9 to 1.5 nM in the cultures (Figure 4, Supplementary Tables 1, 2). Between 10–22 h, the concentrations of methanol were lower than the detection limit [0.24 nM or 7.87 ppbv at 19 °C, (Davie-Martin et al., 2020)], which may indicate that methanol production and its magnitude of diel variation were underestimated in this experiment. The three *m*/*z* signals in Cluster 4^*^ exhibited some intra-cluster variation, but these *m*/*z* signals all increased during the daytime and nearly returned to the dawn concentrations by 24 h. Cluster 6^*^, consisting of 7 *m*/*z* signals, peaked at midday and then returned to the dawn concentrations by 24 h.

### Isostatic VOC production and light-driven VOC accumulation

The clustering of VOCs determined from their concentrations over the diel cycle was influenced by cell division and the light-dark phases (Figure 3). The general decreases in relative VOC concentrations from 10 to 24 h in Figure 3b were caused by accounting for cell densities that increased during that period. In contrast, absolute *m*/*z* signals in Figure 3a (Clusters 1, 3, 5) were maintained at approximately the same concentrations over the entire 24 h period even as the *P. tricornutum* population increased during the nighttime. We call this behavior “isostatic VOC production.” In this case, passive diffusion of VOCs allows them to move from inside the cell, through the cell envelope, and into the bulk medium, where they achieve equilibrium with intracellular VOC concentrations. In the absence of a strong biological sink (i.e., bacterial VOC consumption) (Sun et al., 2011; Beale et al., 2013; Sargeant et al., 2018; Moore et al., 2020) or physical sink (more rapid bubbling that strips VOCs out of the culture or aeration by shaking), VOC concentrations remained stable. As the cell density increased over the nighttime, VOC production continued, albeit at slower cell-specific rates, to maintain the same VOC concentrations in the culture medium. VOCs in Clusters 4 and 6 accumulated rapidly during the light phase until they peaked in concentrations. We call this behavior “light-driven VOC accumulation.” VOCs in Clusters 4 and 4^*^ reached maximum concentrations after 12 h, and VOCs in Clusters 6 and 6^*^ reached maximum concentrations after 6 h. After reaching their maxima, VOC concentrations in Clusters 4^*^ and 6^*^ gradually decreased as the cell density increased.

In addition to relative VOC concentrations, we also examined the absolute concentrations of the 63 *m*/*z* signals over the diel cycle (Figure 4). Individual VOCs accumulated in the cultures to concentrations ranging from 0.03 to 13.68 fmol per cell over the 24 h period, corresponding to rates of VOC production over 24h ranging from 0.5 to 125 fmol (cell·d)^−1^ (determined by summing the 2 h average concentrations over the 24 h period) (Figure 4b). Seven *m*/*z* signals accumulated to concentrations at the high end of the range of 7-13 fmol cell^−1^. Twenty-three *m*/*z* signals reached concentrations exceeding 1 fmol cell^−1^, including *m*/*z* 43.05, 45.03, 47.04, 59.05, 75.04, and 91.03 (Figure 4, Supplementary Table 2). Methanol (*m*/*z* 33.033) concentration varied up to 5-fold over the diel cycle, with cell-specific concentrations of 3–5 fmol cell^−1^ at 0 to 6 h and 22 h but below the limit of detection between 6 and 22 h (Figure 4). Other *m*/*z* signals, including toluene (*m*/*z* 93.07) and styrene (*m*/*z* 105.06), varied up to 2-fold over the 24 h cycle.

**Figure 5:**
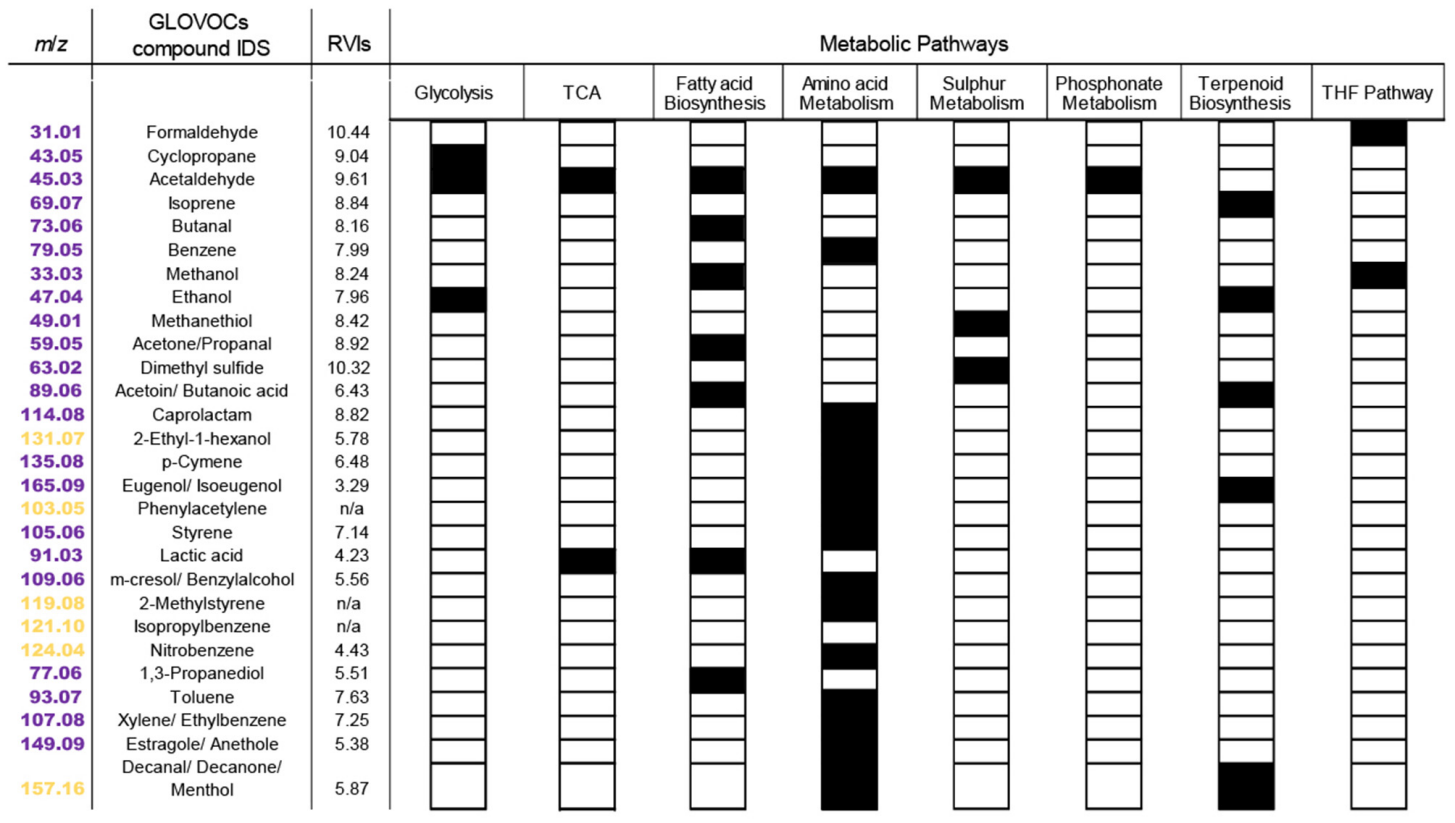
Putatively identified VOCs in KEGG and their metabolic pathways in *P. tricornutum*. In the first column, *m*/*z* signals described in *P. tricornutum* KEGG pathways are colored magenta and plant KEGG pathways are colored yellow. Putative VOC identifications for *m*/*z* signals with two or three isomers are listed with slashes. Each black box indicates the presence of the VOC in the pathway indicated at the top of each column. RVI, relative volatility index.

To further explore VOC temporal dynamics, we focused on clusters determined from cell density-normalized *m*/*z* signal concentrations (Clusters 1^*^-6^*^; Figure 3b) because they represent VOC dynamics related to diel-dependent physiology (Sartori et al., 2021; Ledford and Meredith, 2024), including the cell cycle. Each cluster contained at least one *m*/*z* signal produced at >50 fmol (cell·day) ^−1^. Forty-eight of the 63 *m*/*z* signals were putatively identified using the GLOVOCs database, and twenty-two of those 48 *m*/*z* signals were found in *P. tricornutum* KEGG pathways (Table 2; Figure 5, Supplementary Table 3). Five of the *m*/*z* signals not found in *P. tricornutum* KEGG pathways were present in KEGG pathways in plants. The remaining 21 putatively identified *m*/*z* signals represent metabolites not previously known from *P. tricornutum* or plant KEGG pathways. An additional 15 *m*/*z* signals were unknown (Figure 4, Supplementary Table 3).

**Table 2:** *m*/*z* signal putative IDs determined from GLOVOCs and explored using KEGG.

**Number of *m*/*z* signals**	**Associations in KEGG**
22	Present in KEGG in *P. tricornutum* pathways
5	Present in KEGG in plant pathways
21	Newly reported in *P. tricornutum*, not present in KEGG
15	Unknown

**Figure 6:**
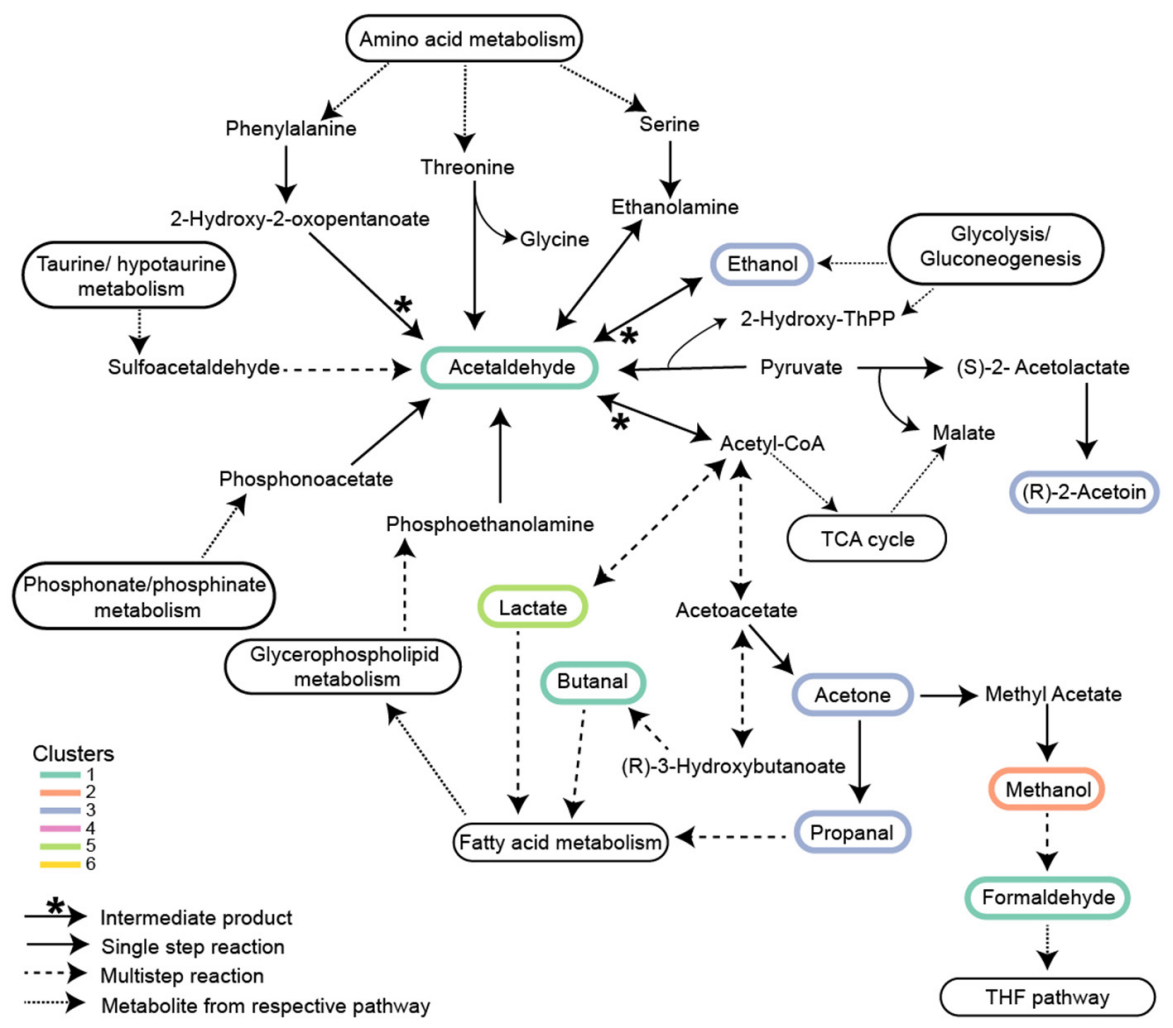
Proposed metabolic network of isostatic VOCs in central carbon metabolism. VOCs in Clusters 1*, 3*, and 5* are involved in Glycolysis/Gluconeogenesis, TCA cycle, and Fatty acid metabolism. Acetaldehyde is involved in nine different reactions and is a central node of intersecting pathways. The association of these isostatic VOCs with methanol in Cluster 2* is shown for clarity. Clusters numbered with an asterisk are from Figure 3b and Figure 4. Solid arrows with an asterisk represent acetaldehyde as an intermediate product; solid arrows represent single-step reactions; dashed arrows represent multistep reactions; and dotted arrows represent metabolites transferred between respective pathways.

Eight *P. tricornutum* metabolic pathways contained the 27 putatively identified VOCs found in KEGG (Figure 5). Some VOCs are metabolites in multiple pathways. For example, acetaldehyde (*m*/*z* 45.03) is a product or substrate in six of the eight VOC pathways (Figures 5, 6), highlighting its pivotal role in the interchange of carbon atoms amongst different cellular processes. The vast majority of VOCs were found in either amino acid metabolism (16 VOCs) or fatty acid synthesis (seven VOCs) (Figure 5). Twenty of the VOCs identified in KEGG were grouped into the isostatic VOC production Clusters 1^*^, 3^*^, and 5^*^ and were distributed across all eight *P. tricornutum* pathways (Figures 3b, 5). Seven VOCs, categorized into the light-driven accumulation Clusters 4^*^ and 6^*^, were associated with amino acid metabolism and secondary metabolite biosynthesis.

We computed the relative volatility index (RVI) for each VOC identified in KEGG. The higher the RVI, the higher the vapor pressure of the compound. RVI may indicate how readily a VOC diffuses across the membrane. RVI will also impact the movement of VOCs into the gas phase and out of the culture in an open system like those used in this study. All of the VOCs identified in KEGG had RVI values >2 and are thus classified as VOCs with ‘high volatility' (Meredith et al., 2023). “Isostatic” VOCs exhibited RVIs in the range of 8–10, whereas VOCs that accumulated in the light had slightly lower RVIs of 5–7 (Figure 5). This difference suggests that diffusive loss of VOCs with high RVIs requires cells to continually replenish their intracellular VOC pools, resulting in the “isostatic production” observed in Clusters 1^*^, 3^*^, 5^*^. VOCs in clusters 4^*^ and 6^*^, with lower RVIs, are less readily diffused out of the culture. These VOCs were upregulated during the light phase, indicating their increased demand during light-driven cell metabolism (Figure 5).

### VOCs in central carbon metabolism and fatty acid metabolism exhibited isostatic production

Isostatic VOCs present in the highest concentrations were part of central carbon metabolism. Most of these highly produced VOCs are metabolic intermediates connecting glycolysis and the TCA cycle (Figure 6). Ethanol (*m*/*z* 47.05) and acetaldehyde (*m*/*z* 45.03), products from glycolysis/gluconeogenesis, can be metabolized into lactic acid (*m*/*z* 91.03) and butanal (*m*/*z* 73.06), which feed into fatty acid metabolism. Similarly, the isomers acetone/propanal (*m*/*z* 59.05) produced by glycolysis feed into fatty acid metabolism. PTR-TOF MS cannot distinguish between isomers of the same mass. Acetone/propanal is generated as a by-product of acetyl-CoA or acetoacetate decarboxylation in plants (Suganuma et al., 1993) and subsequently in *P. tricornutum* can enter either fatty acid metabolism or be converted to methyl acetate and hydrolyzed to methanol (Figure 6). Methanol oxidation to formaldehyde (*m*/*z* 31.01) sources C1 units in the tetrahydrofolate (THF) pathway (Figure 6, Supplementary Table 2). Additionally, *m*/*z* 89.06 corresponding to either acetoin or butanoic acid, depending on the molecular structure, are end products of glycolysis or converted to 2,3 butanediol. Acetoin buffers against pH fluctuation in plants, and its conversion to 2,3-butanediol creates a signal for plant growth (Forlani et al., 1999; Xiao and Lu, 2014). Acetaldehyde, ethanol, and acetone are commonly described as products of stress in plants (Kelsey and Westlind, 2017; Jardine and McDowell, 2023) and algae (Catalanotti et al., 2013; Yu and Li, 2021). For instance, in response to zinc-sulfate deficiency (salt stress), acetaldehyde and ethanol production increased ~3-fold in *Chlamydomonas reinhardtii* compared to replete controls (Van Lis et al., 2017). In our experiments, cells were in balanced, exponential growth under light conditions typical of the summer surface ocean mixed layer. Even at the peak light intensity (400 μE), when photoinhibition was observed, concentrations of acetaldehyde, ethanol, and acetone remained nearly constant (Figure 2). Thus, although VOC production by algae is observed during stress, many VOCs are produced, even in relatively high concentrations, in healthy growing cells (Halsey et al., 2017; Moore et al., 2020).

A goal of this research was to understand how VOC production is related to the diel cycle. The cell-specific production of isostatic VOCs in Clusters 1^*^, 3^*^, and 5^*^ decreased by half over the course of the night. Even at night, some production of these VOCs occurred to maintain their intra-extracellular equilibrium as they were consumed either through metabolism or removal from the system by gentle bubbling. Carbon metabolism in *P. tricornutum* is closely coordinated with the diel cycle (Chauton et al., 2013). Carbon fixation, gluconeogenesis, and fatty acid biosynthesis are upregulated during the early morning when VOCs in clusters 1^*^, 3^*^, and 5^*^ were at their highest concentrations. The TCA cycle and fatty acid beta-oxidation are more highly expressed during the late afternoon (Smith et al., 2016) when VOC production begins to slow. Glycolysis and the TCA cycle are active during the day and night, but the expression of these pathways is partly controlled by their spatial localization in the cytosol, chloroplast, or mitochondria (Smith et al., 2016; Broddrick et al., 2019) where glycolytic isozymes are differentially expressed during the day and night (Chauton et al., 2013; Smith et al., 2016). The amphibolic isozyme, glucose-6-phosphate isomerase, GPI_3, functions in glycolysis/gluconeogenesis in the chloroplast and is upregulated during the day (Smith et al., 2016). In contrast, glucose-6-phosphate isomerase, GPI_1, functions in the cytosol and is most highly expressed at night in tandem with genes in the TCA cycle (Kroth et al., 2008; Chauton et al., 2013). The cell's constant requirement for carbon precursors requires non-stop activity of central carbon metabolism. Consequently, isostatic VOCs in Clusters 1^*^, 3^*^, and 5^*^ are produced continuously, at levels that depend on the activities of their respective pathways at different times of the day.

### VOCs in amino acid metabolism and secondary metabolite biosynthesis accumulated during the light phase

**Figure 7:**
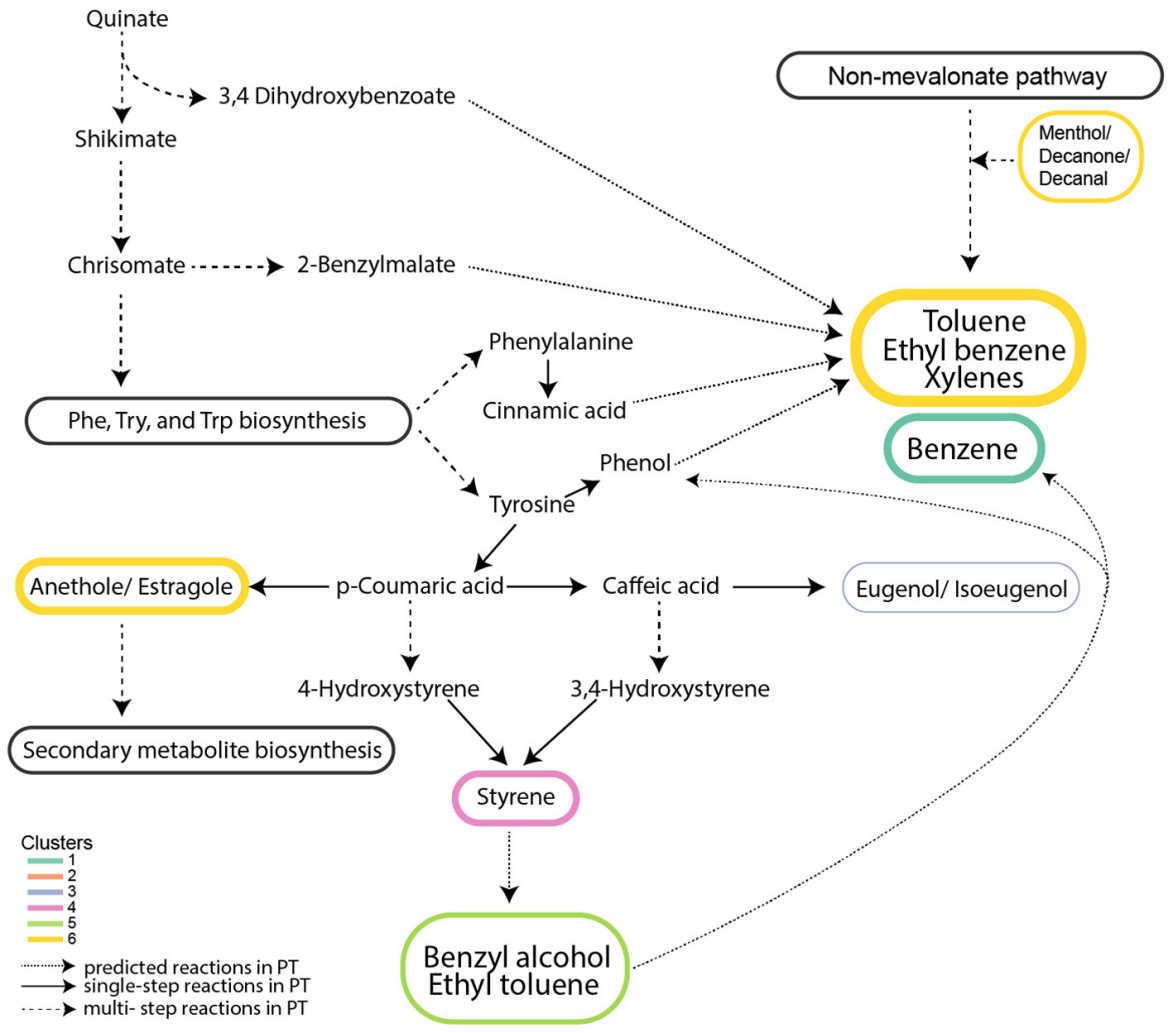
Metabolic network of VOCs exhibiting light-driven accumulation. VOCs from clusters 4* and 6* are linked to aromatic amino acid metabolism and secondary metabolite biosynthesis. Clusters numbered with an asterisk are from Figure 3b and Figure 4. Solid arrows represent a single-step reaction; dashed arrows represent a multistep reaction; and dotted arrows represent predicted metabolites from respective pathways.

VOCs in Clusters 4^*^ and 6^*^ accumulated during the light phase and are produced from aromatic amino acid metabolism and the biosynthesis of secondary metabolites (Figures 5, 7). In Cluster 4^*^, styrene (*m*/*z* 105.06) is a by-product of p-coumaric acid and caffeic acid metabolism in secondary metabolite biosynthesis (Figure 7). Secondary metabolite production and amino acid biosynthesis co-occur with protein and carbohydrate accumulation in plants (Liebelt et al., 2019). Benzenoid compounds from Cluster 6^*^, including toluene (m/z 93.07) and the isomers ethylbenzene/xylenes (m/z 107.08), are produced by aromatic amino acid metabolism. Their production during the daytime is consistent with the daytime upregulation of biosynthesis genes for tyrosine and phenylalanine (Smith et al., 2016). Also, in Cluster 6^*^, the isomers anethole/estragole (*m*/*z* 149.06, Figure 7), metabolized from p-coumaric acid, protect against high light stress and have antimicrobial activity in plants (Koeduka et al., 2009).

The roles of mono-aromatic benzenoids, including benzene, toluene, ethylbenzene/xylenes, and styrene, in algae remain largely unknown. They have been proposed to serve as precursors for aromatic compound synthesis in plants (Misztal et al., 2015). In the cytosol of plants, approximately 30% of the carbon derived from photosynthesis is directed toward phosphoenolpyruvate and erythrose-4-phosphate synthesis, which feed the shikimate pathway (Maeda and Dudareva, 2012) used for biosynthesis of aromatic amino acids phenylpropanoid and benzenoid production (benzyl alcohol, benzaldehyde, benzyl benzoate, methyl benzoate, phenylacetaldehyde, and phenyl ethanol) (Orlova et al., 2007). Alternatively, some benzenoids may be products of protein degradation (Dudareva et al., 2013). Suppression of benzenoid compound production in plants affected their morphology and decreased transport of auxin, a hormone that facilitates cell division and growth (Orlova et al., 2007). It is possible that benzenoids have roles associated with the cell cycle in diatoms. Unexplained is why benzene (m/z 79.05) from Cluster 1^*^, likely produced from aromatic amino acid metabolism, appears to be isostatically produced rather than showing accumulation during the light phase as the other VOCs functioning in aromatic amino acid metabolism pathways.

### Methanol (*m/z* 33.03) temporal behaviors may reflect *P. tricornutum* cell cycle dynamics

**Figure 8:**
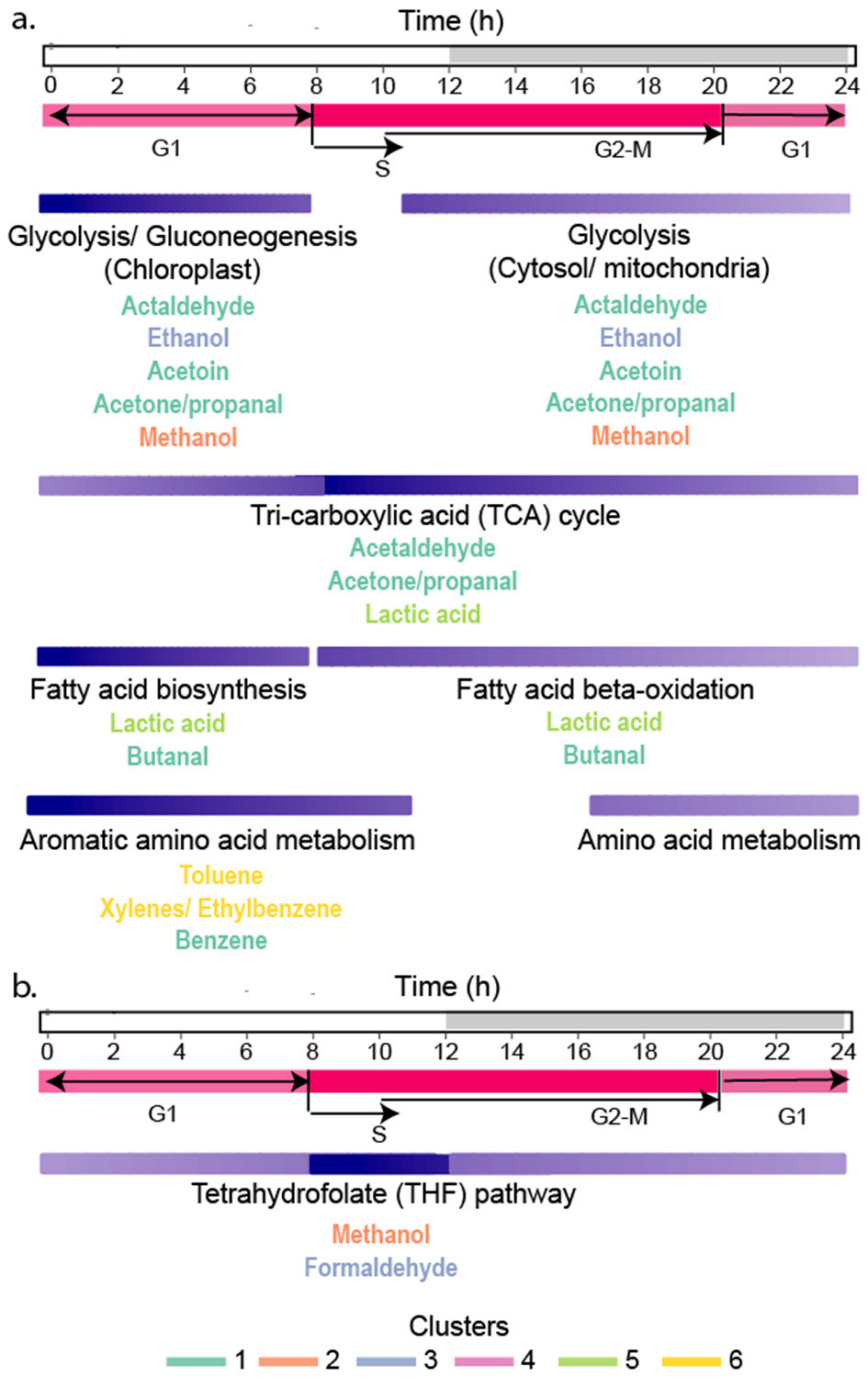
Relationships between VOC metabolism and the cell cycle in *P. tricornutum*. **(a)** Activities of pathways involved in VOC production are indicated by the shading of the purple bars (dark purple shows periods of greatest activity and most VOC accumulation, and light purple is lower activity and VOC production). Purple bars for each metabolic pathway are aligned with the diel cycle at the top, and cell cycle phases are shown in pink. **(b)** Metabolism of methanol by the THF pathway was most active during S-phase, causing rapid methanol *depletion* in culture. The font color of each VOC indicates its grouping into each cluster.

The temporal pattern exhibited by methanol (m/z 33.033) (Cluster 2^*^; Figures 3, 4) suggests its production is strongly associated with the diatom's mitotic cell cycle. In *P. tricornutum*, the S-phase, DNA synthesis, begins 2 h before dark (Figure 8) (Brzezinski, 1992; Kim et al., 2017). In our experiment, methanol was high in concentration during the daytime and dropped below the limit of detection 2 h before dusk, aligning with S-phase (Figure 8). This temporal correspondence suggests the intracellular methanol pool was rapidly consumed to generate C1 equivalents for nucleic acid biosynthesis during DNA synthesis. Methanol can be oxidized to formaldehyde (Figure 6) and spontaneously react with tetrahydrofolate (THF) to form 5,10-methylene-tetrahydrofolate, which donates C1 units for nucleotide biosynthesis (Nuccio et al., 1995; He et al., 2020; Bou-Nader et al., 2021). Methanol concentrations increased as the cells entered another growth phase. Formaldehyde was grouped in Cluster 1^*^, which showed little change over the diel cycle, possibly reflecting the constant need for C1 units in RNA biosynthesis and energy production throughout the cell cycle.

### Isoprene, DMS, methanethiol, and their diel patterns

Isoprene (m/z 69.07; Cluster 1^*^); dimethyl sulfide (DMS, m/z 63.02; Cluster 3^*^), and methanethiol (m/z 49.01; Cluster 3^*^) were among the VOCs that were isostatically produced. These metabolites are commonly observed during cell stress, such as high light (Van Rijssel and Gieskes, 2002; Halsey et al., 2017; Steinke et al., 2018) and grazing (Ploug and Grossart, 2000; Fisher et al., 2020). Isoprene exhibits antioxidant properties (Pollastri et al., 2021; Zhao et al., 2024), but the balanced growth conditions in our experiments likely did not induce photic stress, which could explain low isoprene, DMS, and methanethiol concentrations. Daytime maxima in the concentrations of these compounds have been observed in other diatom cultures (Kameyama et al., 2011), coral reefs and the open ocean (Galí et al., 2013; Davie-Martin et al., 2020; Masdeu-Navarro et al., 2024). However, the isostatic production of isoprene, DMS, and methanethiol observed in this study suggests that other factors besides daily changes in light intensity, such as bacterial heterotrophic consumption (Boden et al., 2011; Moore et al., 2022), daytime grazing (Ng and Liu, 2016), photooxidation (Kieber et al., 1990; Masdeu-Navarro et al., 2024), and variable mixing depth (Halsey and Giovannoni, 2023) are likely to underlie daily fluctuations in VOC concentrations.

VOCs measured in this study exhibited diel variations associated with changes in the light and dark reactions of photosynthesis that shift over hourly timescales (Halsey et al., 2013). The culture experiment was limited to 24 h to ensure that cells remained in exponential growth. The observed VOC dynamics are expected to repeat if cell physiology is maintained via dilution with fresh media and light regime replicated. Subtle differences in the temporal dynamics of VOCs produced from metabolic pathways common across phytoplankton are hypothesized to be associated with species-specific regulation of their metabolic pathways. An important goal will be to understand taxonomic variation in VOC production patterns, which could help describe surface ocean VOC accumulation depending on community composition and may be predictable given sufficient understanding of the genetic components involved.

## Summary

Relating in-water biological VOC production to atmospheric chemistry processes, such as ozone formation and depletion and condensation reactions that form SOA, is challenging because of the complexity of compounds involved, diversity of biological sources and sinks, and a lack of knowledge about the conditions that influence VOC accumulation in the surface ocean. Here, we showed that VOCs were produced throughout the diel cycle in a cultured diatom, with the highest rates of VOC production occurring during the daytime and involved in amino acid and fatty acid biosynthesis. Light intensities were saturating at midday, causing slight photoinhibition, but the patterns of VOC production were more strongly associated with diel shifts in cell physiology than stress responses. We documented strong VOC accumulation during the morning hours, consistent with the requirement for photosynthesis to produce sufficient organic carbon to support cell needs for growth and division. Nighttime metabolism includes cell division, protein synthesis, and pre-dawn preparation for photosynthesis. Thus, nighttime VOC production was caused by the ongoing flux of carbon through glycolysis, the TCA cycle, and fatty acid beta-oxidation. Positive air-sea emissions (into the atmosphere) of benzene, toluene, and xylenes (ethylbenzene + xylene) can be at least as large as DMS emissions, indicating these benzenoids should be recognized as major contributors to atmospheric chemistry and SOA formation. Our results show these benzenoids primarily originate from daytime photosynthetic processes in the model diatom *P. tricornutum*.

Diel VOC production dynamics give a fundamental understanding of the first steps in VOC accumulation in the surface ocean. The complex biological and abiotic processes that next act upon in-water VOCs will impact their concentrations in ways that are specific to each VOC. To understand the chemical composition of the surface ocean and overlying atmosphere will require an interdisciplinary approach to incorporate into next generation ocean-atmosphere models the dynamic roles of biological VOC metabolism, chemical transformations, and physical transport.

## Data Availability

The original contributions presented in the study are included in the article/Supplementary material, further inquiries can be directed to the corresponding author.
