# Supplementary material for: Diatom volatile organic compound production is driven by diel metabolism and the cell cycle
Source: Front Microbiol. 2025 Oct 6;16:1620542. doi: 10.3389/fmicb.2025.1620542 (PMC12535992; doi:10.3389/fmicb.2025.1620542)
Supplement: Supplementary file 4 [file Image_1.pdf]

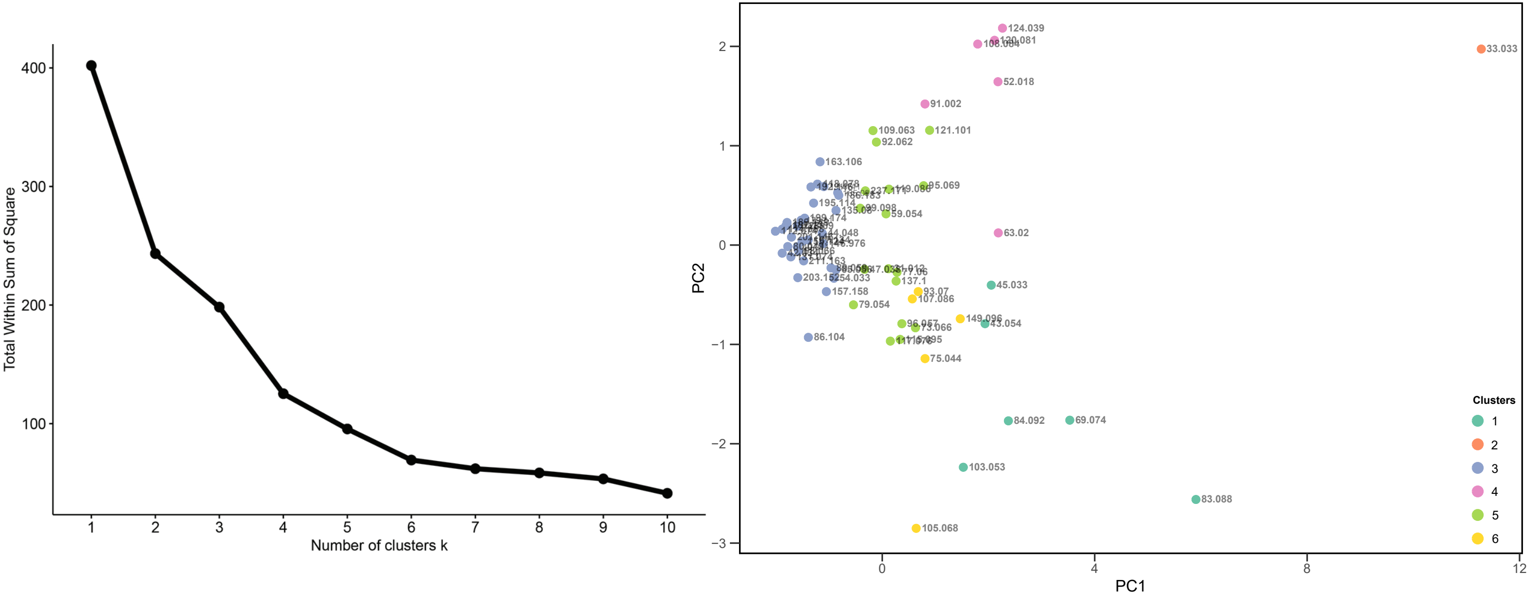

Supp. Figure 1A: (left) Elbow plot showing number of clusters to the total within the sum of squares, i.e., average squared distance from the centroid to each *m/z* B-spline coefficient when VOC concentrations were normalized to their maximum values. (right) PCA plot showing the clusters when k = 6.

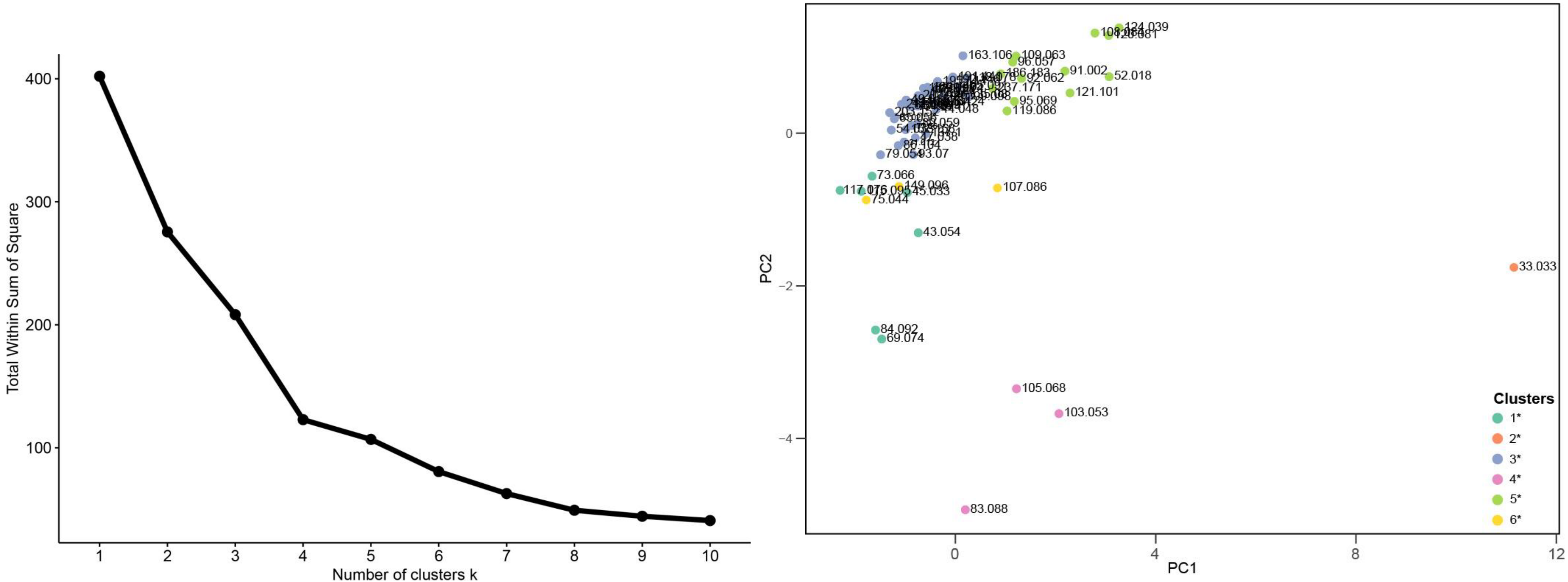

Supp. Figure 1B: (left) Elbow plot showing number of clusters to the total within the sum of squares, i.e., average squared distance from the centroid to each *m/z* B-spline coefficient when VOC concentrations are normalized to cell density and then to their maximum values. (right) PCA plot showing the clusters when k = 6.
